# A systematic review and mixed-methods synthesis of the experiences, perceptions and attitudes of prison staff regarding adult prisoners who self-harm

**DOI:** 10.1192/bjo.2022.70

**Published:** 2022-06-06

**Authors:** Thomas Hewson, Kerry Gutridge, Zara Bernard, Kathryn Kay, Louise Robinson

**Affiliations:** Faculty of Biology, Medicine and Health, University of Manchester, UK; and North West School of Psychiatry, Health Education England, University of Manchester, UK; Centre for Women's Mental Health, University of Manchester, UK; Greater Manchester Mental Health NHS Foundation Trust, UK; Greater Manchester Mental Health NHS Foundation Trust, UK; Division of Psychology and Mental Health, University of Manchester, UK; and Lancashire and South Cumbria NHS Foundation Trust, UK

**Keywords:** Self-harm, suicide, prison, staff, attitudes

## Abstract

**Background:**

Self-harm, including suicide, is common among prisoners. Staff attitudes and perceptions regarding self-harm may affect quality of care and patient safety.

**Aims:**

To systematically review the experiences, perceptions and attitudes of staff in adult prisons regarding self-harm.

**Method:**

Systematic searches of EMBASE, Medline, PsycINFO and CINAHL databases were conducted, and supplemented by hand-searching and grey literature review, to identify relevant English-language articles published since the year 2000. Articles were screened by two authors and evaluated with standardised quality appraisal tools. Qualitative data were analysed thematically, whereas quantitative data were narratively synthesised because of high study heterogeneity.

**Results:**

Two thousand articles were identified, of which 32 were included, involving 6389 participants from five countries. Most studies were moderate (*n* = 15) or poor (*n* = 10) quality, and seven were rated as good quality. Staff frequently witnessed self-harm and described multiple perceived risk factors and causes of this. Perceptions that self-harm is ‘manipulative’ or ‘attention-seeking’ were associated with hostility toward prisoners and lower quality of care. Perceived barriers to preventing and managing self-harm included low staffing levels, prison environments and culture, poor staff confidence and insufficient training. The importance of multidisciplinary teamwork and building staff–prisoner relationships were highlighted. Staff occasionally experienced intense psychological reactions to self-harm, which resulted in adaptive or maladaptive coping that influenced their capacity to care.

**Conclusions:**

There are mixed attitudes and perceptions toward self-harm among prison staff. Further training, support and resources are required to protect staff's well-being and improve self-harm prevention and management in prisons.

The Ministry of Justice defines self-harm as ‘any act where a prisoner deliberately harms themselves irrespective of the method, intent, or severity of any injury’.^1^ Both self-harm and suicide are more common among prisoners compared with the general population.^2–5^ In the 12 months to September 2021, there were 52 726 recorded incidents of self-harm in English and Welsh prisons.^6^

Self-harm causes significant distress among prisoners, their families and prison staff, and has significant costs to the National Health Service and prison services.^7–9^ People who self-harm are at increased risk of physical and mental morbidity, premature mortality and suicide.^10,11^ The effective prevention and management of self-harm is an important aspect of national suicide prevention strategies and the Prison Service Competency and Qualities Framework.^12,13^

## Experiences, perceptions and attitudes of clinical staff regarding self-harm

Self-harm is a common presentation to clinical services.^14^ Although many healthcare professionals support people who self-harm, negative staff responses have been described across settings, including emergency departments, general medical and psychiatric environments.^15–21^ These reactions include feelings of irritation, anger and antipathy.^22,23^ Healthcare staff may also hold more hostile attitudes toward people who self-harm compared with other patients,^22^ and lack confidence in supporting them, reporting feeling helpless and ill equipped.^15,23,24^ These attitudes and feelings can arise from poor understanding, work stress, stigma, being personally emotionally affected from witnessing self-harm and psychological defence mechanisms.^22,25^

Negative staff attitudes toward self-harm can result in their hostility toward, and distancing from patients, poor empathy and stigmatisation.^26^ This can affect quality of care, increase a person's risk of future self-harm and deter them from treatment and/or engagement with clinical services.^26–30^ Conversely, demonstrating respect and kindness can diminish feelings of shame and instil hope,^28^ and potentially reduce self-harm.^25^

## Aims and context of this research

Prisoners who self-harm interact with both healthcare and prison staff. Understanding the experiences, perceptions and attitudes of these staff is important in understanding the care delivered, and any facilitators or barriers to reducing self-harm in prison. Prison staff's experiences of managing self-harm may also provide insights into its effect on their well-being.

We aimed to systematically review the research literature to answer the question, ‘What are the experiences, perceptions, and attitudes of prison staff regarding adult prisoners who self-harm?’. To the best of our knowledge, no prior review of this literature has been published.

## Method

We sought to collate all relevant studies of prison staff's experiences of, and perceptions or attitudes toward, self-harm in prisons for adults. Since this study is a systematic review of previously published research, ethical approval was not necessary. The protocol for this review is registered on the International Prospective Register of Systematic Reviews (PROSPERO; identifier CRD42020190618).

### Search strategy

Electronic databases (EMBASE, Medline, PsycINFO and CINAHL) were searched on 24 April 2020 for relevant international literature. The search was restricted to articles published since the year 2000, to determine current, rather than historical, attitudes, perceptions and experiences of prison staff relating to self-harm. Search terms were agreed between authors and ‘exploded’ or searched as MESH terms, to identify further relevant terminology (Supplementary Appendix 1 available at https://doi.org/10.1192/bjo.2022.70). Grey literature was additionally consulted on 18 May 2020, including reviewing the first 100 outputs from Google and Google Scholar, searching the Open Grey database, and reviewing relevant organisations websites such as The Howard League, Ministry of Justice, Independent Advisory Panel on Deaths in Custody, and Prison Reform Trust (Supplementary Appendix 2). Reference lists of relevant studies were hand searched.

Criteria for inclusion were as follows: participants were staff of any role or grade, working within prisons for adults, whose experiences of, perceptions of, or attitudes toward, self-harm were assessed with any quantitative or qualitative method. The Ministry of Justice definition of self-harm was used.^1^ All studies must have been published in the English language. All publication types were considered except for expert opinion papers, systematic reviews and editorials. Studies conducted exclusively in juvenile correctional settings and young offender institutes (YOIs) were excluded unless they provided data specific to staff's experiences with adult prisoners. This is because age is an influencing factor in determining staff attitudes toward self-harm,^31^ and staff working in YOIs often manage both adolescents and adults. Studies conducted in secure mental health facilities or community settings were additionally excluded.

### Study selection and quality assessment

Following the deletion of duplicates and non-English texts, all articles were independently screened for eligibility by two authors (T.H. and K.K.). Any disagreements regarding the exclusion of articles were resolved by consulting a third reviewer (L.R.), who made the final decision. The quality of each study was also independently assessed by two authors, using the following standardised quality appraisal tools: the Critical Appraisal Skills Programme Qualitative Checklist for qualitative studies (T.H. and Z.B.);^32^ the National Heart, Lung, and Blood Institute Quality Assessment Tool for Observational, Cohort and Cross-sectional Studies (T.H. and L.R.)^33^ and the Mixed Methods Appraisal Tool for mixed-methods study designs (T.H. and K.G.).^34^ Overall quality ratings of good, moderate or poor were assigned to each study. Any disagreements regarding quality ratings were resolved by discussion to achieve consensus.

### Data extraction and synthesis: qualitative data

A standardised template was designed to collect relevant data from eligible studies. Relevant qualitative data from all studies were imported verbatim into NVivo 12 for Windows software (QSR International, Ltd., Australia; https://www.qsrinternational.com/nvivo-qualitative-data-analysis-software/home), and independently analysed by two authors (T.H. and Z.B.).^35^ A thematic synthesis was performed with inductive methods, whereby codes were derived directly from the data.^36^ Each line of text was read, interpreted and coded according to its content and meaning. All codes were then compared between authors and analysed for similarities and differences. The codes were subsequently amalgamated into hierarchical themes, and new codes were created to group initial codes together. Final broad themes were agreed by both authors and interrogated by a third author (K.G.).

The small number of quantitative studies and the heterogeneity of methodology used between studies precluded the use of meta-analysis. Instead, a narrative synthesis of quantitative data was conducted following the framework described by Petticrew and Roberts.^37^ Studies reporting quantitative data were initially categorised by types of outcome measure and the specific aspects of staff's experiences or perceptions of, or attitudes towards, self-harm being examined. Within-study analysis was conducted by describing the main findings from each study and significant methodological aspects. Cross-study synthesis was subsequently performed by summarising the overall results and similarities or differences between studies, and taking account of variations in research design.

## Results

**Fig. 1:**
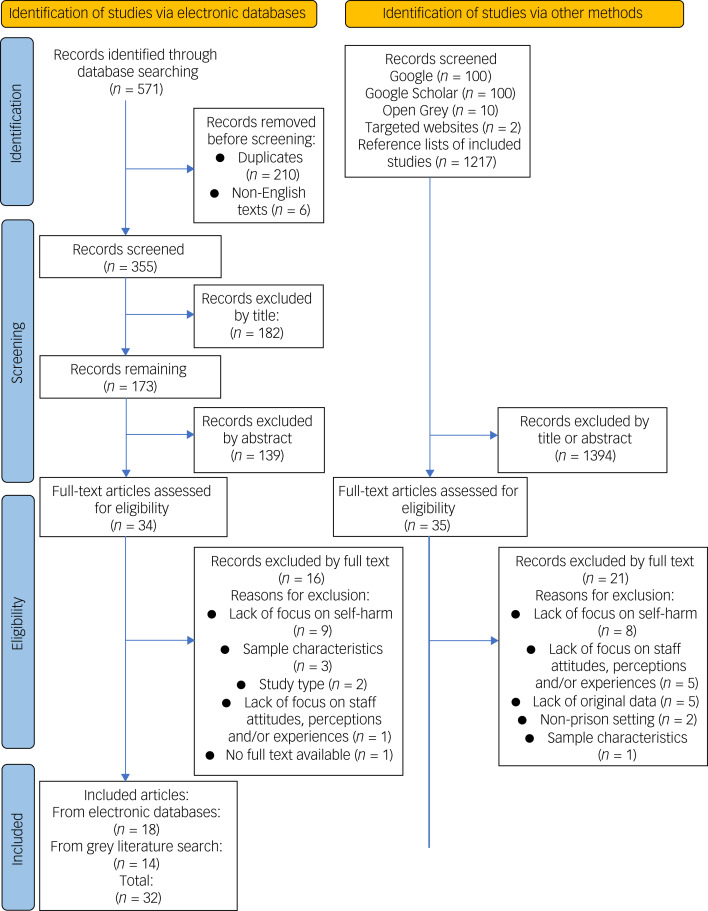
Preferred Reporting Items for Systematic Reviews and Meta-Analyses (PRISMA) flow diagram.

A total of 571 articles were identified from electronic databases and 1429 articles were retrieved from other sources (*n* = 2000) (Fig. 1). Following removal of duplicates and non-English texts, 1784 articles were screened by title and abstract. The full texts of 69 articles were assessed for eligibility, of which 32 met criteria for inclusion in the review (Table 1). Reasons for the exclusion of articles are detailed in Supplementary Appendix 3.

**Table 1:** Summary characteristics of included studies

Study (year) country	Data type	Data collection methods	Setting	Sample
Bantjes et al (2017)^38^ South Africa	Qualitative	Semi-structured face-to-face interviews	One medium and one maximum security correctional centre in Cape Town	Ten health professionals
Howard League for Penal Reform (2017)^39^ UK	Qualitative	Interviews and focus groups	Eight prisons ranging from Category A to C, and six NHS Trusts	Unknown sample size of multidisciplinary prison staff, including managers, nurses, psychologists, psychiatrists, safer custody officers and independent clinical reviewers
Kenning et al (2010)^40^ UK	Qualitative	Semi-structured interviews	Women's prison in North-West England	15 prison staff, including eight discipline staff, five healthcare staff and two governors
Ludlow et al (2015)^41^ UK	Qualitative	Semi-structured interviews, focus groups, and intensive participant observation	Five prisons, including two male local Category B prisons, one male adults/young offenders, one male adult and young offenders and one female adults/ young offenders prison	47 multidisciplinary prison staff (interviews), five staff focus groups, and staff members participating in 15 ACCT reviews
Marzano (2007)^42^ UK	Qualitative	Semi-structured interviews	Local male prison in South-East England	38 prison staff, including 15 prison officers, 15 healthcare staff and eight specialists
Marzano et al (2013)^43^ UK	Qualitative	Semi-structured face-to-face interviews	Male prison in South-East England for adult and young offenders	30 correctional staff, including 15 officers, 13 nurses and two doctors
Moore et al (2010)^44^ UK	Qualitative	Semi-structured interviews	Women's remand prison	Seven prison officers and eight clinically qualified staff
Ramluggun (2013)^45^ UK	Qualitative	Semi-structured interviews, questionnaire and documentary evidence review	Category B adult male prison	37 prison staff, including nursing staff, prison officers, managers and the governor
Rivlin (2007)^46^ UK	Qualitative	Semi-structured face-to-face interviews	Category B adult male prison	13 prison staff, including two nurses, one psychologist and ten prison officers
Rivlin (2010)^47^ UK	Qualitative	10-week period of participant observation and semi-structured interviews	Category B adult male prison	13 prison staff, including two nurses, one psychologist and ten prison officers
Short et al (2009)^48^ UK	Qualitative	Semi-structured interviews	Women's prison in North England for adult and young offenders	13 prison staff, including eight prison officers and five healthcare staff
Smith et al (2019)^9^ USA	Qualitative	Semi-structured telephone interviews	Various USA state prisons	41 prison staff, including directors, administrators, psychiatrists and psychologists
Sweeney et al (2018)^49^ UK	Qualitative	Semi-structured interviews	Category B male prison in Yorkshire	Nine prison officers with experience of working with offenders who had engaged in suicidal behaviour
Tait (2011)^50^ UK	Qualitative	Interviews, participant observation and questionnaire	One rural local prison for females, and one urban local prison for males	24 male and 21 female prison officers with direct responsibilities for supervising prisoners
Walker et al (2016)^51^ UK	Qualitative	Semi-structured face-to-face interviews	Three closed-category women's adult English prisons	14 prison staff, including ten officers, one governor and three healthcare staff
Walker et al (2017)^52^ UK	Qualitative	Semi-structured face-to-face interviews	Three closed-category female adult English prisons	14 prison staff, including ten officers, one governor and three healthcare staff
Callahan (2004)^53^ USA	Quantitative	Survey	A large mid-Western state Department of Corrections	1877 correctional officers attending mental health training
Cassidy and Bruce (2019)^54^ UK	Quantitative	Self-report survey	One prison (category and gender not stated)	211 correctional staff
Garbutt and Casey (2015)^55^ UK	Quantitative	Survey	Two high-security UK prisons	97 prison employees, including officers, managers, psychologists, offender supervisors and chaplains
Ireland and Quinn (2007)^56^ UK	Quantitative	Survey	Prison service college	162 prison officers from various establishments
Pannell et al (2003)^30^ Australia	Quantitative	Surveys	1 male prison and 1 male remand facility in South Australia	76 correctional officers
Slade and Lopresti (2013)^57^ UK	Quantitative	Survey	Six English prisons: two category B male local prisons, one male category C prison, one female closed prison and two young offender closed establishments	281 prison officers, custodial managers, governors and operational support grades
Smith and Kaminski (2010)^58^ USA	Quantitative	Survey	473 state correctional facilities encompassing multiple security levels and all genders	230 correctional staff, including 205 mental health staff, 13 managerial roles and 12 unspecified positions
Sousa et al (2019)^59^ Portugal	Quantitative	Survey	Three prisons in North Portugal	176 prison officers
Wood-Schultz (2012)^60^ USA	Quantitative	Surveys	American Correctional Association	84 members of the American Correctional Association, including correctional personnel, mental health workers and medical personnel
Wright et al (2006)^61^ UK	Quantitative	Surveys	UK Prison Service	49 prison officers identified as having been closely involved in a prisoner suicide
Cresswell et al (2018)^62^ UK	Qualitative and quantitative	Survey, interviews and documentary analysis	One women's prison in England	68 prison staff for survey and 13 for interviews
DeHart et al (2009)^63^ USA	Qualitative and quantitative	Survey and semi-structured telephone interviews	14 correctional facilities across South Carolina, including all security levels and both male and female prisons	54 (survey) and 18 (interview) correctional mental health professionals
Liebling et al (2005)^64^ UK	Qualitative and quantitative	Surveys, interviews and review of institutional performance data and suicide figures	12 UK prisons, including two male local prisons, two closed female remand and training prisons, four male local and remand prisons, two male closed YOI and remand centres, one closed female remand and training prison and one male local and remand prison	1301 and 1365 multidisciplinary prison staff completed surveys in 2002 and 2004, respectively
Ward and Bailey (2011)^65^ UK	Quantitative and qualitative	Staff training records, process mapping events, surveys and semi-structured interviews	UK women's prison	68 prison staff (surveys) and 13 prison staff (interviews), including discipline staff and multidisciplinary groups involving education, psychology, healthcare, drug workers and chaplaincy
Ward and Bailey (2013)^66^ UK	Quantitative and qualitative	Staff training records, process mapping events, surveys and semi-structured interviews	UK women's prison	68 prison staff (surveys) and 13 prison staff (interviews), including discipline staff and multidisciplinary groups involving education, psychology, healthcare, drug workers and chaplaincy
Ward (2014)^67^ UK	Quantitative and qualitative	Survey, interviews and process-mapping	Women's prison in England	68 prison staff (survey) and 13 prison staff (interviews), including prison officers and multidisciplinary groups

NHS, National Health Service; ACCT, Assessment, Care in Custody and Teamwork processes; YOI, young offender institute.

### Studies included in the review

Of the 32 studies included, most were conducted in the UK (*n* = 24). Other countries represented included South Africa (*n* = 1), the USA (*n* = 5), Australia (*n* = 1) and Portugal (*n* = 1). Seventeen studies collected qualitative data, nine collected quantitative data, and six used mixed methods. Although most studies (*n* = 21) utilised mixed samples of professional groups working in prisons, seven focused exclusively on prison officers and two included only health professionals. In two studies, ‘prison staff’ were referred to without specifying which group/s this included.^54,64^ The total number of participants across all studies, accounting for duplicate samples, equalled 6389 prison staff, although one study did not report sample size.^39^

### Qualitative data synthesis

Twenty two papers containing qualitative data were included in the review. Four broad themes and 12 subthemes emerged from the analysis.

### Theme 1: prison staff's experience of preventing and managing self-harm

#### Subtheme 1.1: high frequency and variety of self-harm in prisons

Witnessing a high frequency of self-harm was reported by prison staff in seven studies, described as occurring ‘all the time’ and ‘every time’ staff complete shifts.^9,38,42–44,62,63^

‘I would say frequently, so every time I have a session at the prisons, there is at least one person that exhibits such behaviour or ideation’ (Officer).^38^

Staff witnessed numerous types of self-harm, with cutting being the most mentioned.^9,38,42–44,62,63^

#### Subtheme 1.2: staff's perceived capacity and ability to manage self-harm

Despite the high frequency of self-harm witnessed, prison staff frequently expressed low confidence in understanding, preventing and managing self-harm.^9,41,48,49^ This resulted in them feeling ‘helpless’ and ‘useless’, particularly if the self-harm was repetitive.^41–44,48,64^

‘There was nothing, I mean, you could do for him, I mean, make(s) you feel sort of useless’ (Healthcare staff).^43^

To improve their confidence and skills, many prison staff highlighted a need for more self-harm training,^9,38,39,41,45,48,49,51,52,63,65,67^ although some emphasised learning ‘on the job’.^41,64^

^‘^The [prison officer's] frustrations are they don't get it [self-harm], and I think if you help and guide them and help them get it, or at least help them understand why that is, it becomes less anxiety provoking for them and for the women then as well’ (Unspecified staff role).^51^

Various occupational factors hindered staff's perceived abilities to care for self-harm. Inconsistent and insufficient staffing was felt to interrupt consistency of care and limit the identification and management of relevant risk factors. Staffing problems also reduced opportunities for multidisciplinary input for prisoners who self-harm, and decreased the time available for staff–prisoner engagement.^9,38,39,41–43,48,49,64^

^‘^Staffing is the main issue for it all. Suicide rates would come down if there was more staff … ’ (Officer).^49^

Staff described various challenges caring for people who self-harm in prisons, which were portrayed as ‘anti-therapeutic’ and ‘punitive’ settings.^38,63,64^ Staff frequently reported tensions between their ‘security’ and ‘care’ responsibilities, and the prison ‘regime’ was positioned as taking priority over healthcare.^9,41,43,48,50,64^ In contrast, in one establishment with low rates of self-harm, staff felt that their roles of ‘carer’ and ‘security officer’ were well integrated.^46,47^ A prison culture was sometimes reported whereby staff felt shamed from outwardly expressing concern for prisoners who self-harm,^42,64^ although this was less apparent among female staff.^64^

^‘^There is (.) stigma attached to being a, ehm, a care bear, they call them in here – in the Prison Service – officers who care too much … ’ (Officer).^42^

Staff generally felt more confident and able to manage self-harm when working in multidisciplinary teams. This allowed them to draw on broad skills and knowledge, resolve prisoners’ issues more quickly and achieve greater consistency of care.^39,41,42,54^ However, staffing and communication difficulties sometimes prevented effective teamwork, in addition to conflicting team perspectives and complex team structures.^38,41,45,63^

#### Subtheme 1.3: emotional effects of self-harm on prison staff

Numerous reactions to self-harm were described by prison staff, varying from frustration and feeling attacked to feeling ‘sad’ and ‘touched’.^38,42,45,50,52,63,64^ Many staff reported stress, anxiety, ‘burnout’ and ‘exhaustion’, which negatively affected their attitudes and care toward prisoners.^9,38,41–43,49,52,63,67^ Some staff felt ‘traumatised’ from witnessing self-harm and developed features of post-traumatic stress disorder,^39,41,43,49,52,67^ and others reported that they became clinically depressed and self-harmed themselves.^42,49^

‘They [prison staff] used to phone me up at home in floods of tears because they kept hearing a prison chewing through her skin, and that's all they could hear’ (Governor).^52^

Many staff were fearful of being blamed or punished for prisoners’ self-harm, especially in Coroner's court.^41–43,45,52,64,67^

‘ … Nobody wants to get entirely involved in such a situation. Just in case that person try and hang themselves. Nobody wants to be taken to the coroner's inquest … ’ (Healthcare staff).^43^

Various coping strategies for dealing with the emotional effects of self-harm were described. Some staff viewed self-harm pragmatically as ‘part of the job’.^9,42,49,52^ Perceiving suicide as a rational ‘choice’ or ‘determined effort’ was suggested to allay feelings of guilt and responsibility.^9,41,42,63^ Other coping strategies included using humour,^42,43,49^ taking ‘time out’ following incidents,^52^ seeking support from colleagues^39,41,49,52,63^ and maintaining a good work–life balance.^42,49,52^ Some staff described maladaptive coping methods, such as alcohol intake and avoidance behaviour.^42,43,49,50,52^

Staff frequently reported needing more practical and emotional support from managers, including greater positive recognition for effectively dealing with self-harm and less emphasis on ‘the failures’.^39,41,50,64^ Several support mechanisms were described including post-incident debriefs and access to formal medical support, but many staff avoided engaging with formal support systems.^39,42,49,52^ This was because of concerns about preserving their masculinity, confidentiality and avoiding being touched by stigma, as well as perceptions that accessing support represents weakness or poor coping.^42,45,52,67^ Staff also felt that opportunities for support following self-harm incidents were limited by pressures to maintain their duties and ‘keep the regime going’.^41,43,67^

^‘^The whole ethos in this prison seems to be IT'S HAPPENED. GET OVER IT. CARRY ON because we've got to, we've got to let them out for feeding or, or exercise, or something’ (Officer).^43^

### Theme 2: attitudes and perceptions of prison staff regarding self-harm

#### Subtheme 2.1: perceived risk factors for self-harm

Mental disorder was reported as a risk factor for self-harm by prison staff across 12 studies.^30,38,40–42,44,45,48,62–64,67^ Substance misuse was most commonly mentioned,^30,39,41,42,48,64^ although some staff referred to psychosis, schizophrenia, depression and personality disorder.^30,38,41,42,48^ Mental disorder was frequently associated with repeated self-harm,^40,42,48,62,67^ and seen as being more legitimate or ‘genuine’ relative to other risk factors.^42,43,62^ When self-harm was associated with mental disorder, prison officers often felt incapable of adequately supporting the prisoner.^38,42,48^

‘You'll find for the prolific self-harmers that they've got mental issues … ’ (Officer).^48^

Negative childhood experiences and/or past abuse were highlighted as risk factors for self-harm by prison officers and healthcare staff.^30,42,47,48^ Staff across several studies recognised stressors within prison environments including boredom, isolation, loneliness, bullying and borrowing between prisoners,^30,38–42,45,48,49,64,66,67^ which some officers viewed as self-inflicted, illegitimate reasons for self-harm.^42^

‘It's like, you are doing that [self-harm] because you are, you are moaning about your situation. But you put yourself in that situation … Take responsibility. Take responsibility for your actions, and just deal with it. Deal with your time’ (Officer).^42^

Arrival into prison was described as a particularly vulnerable period,^39,41,64,67^ along with status change from remand to convicted and receiving very short or long custodial sentences.^41,42^ Other recognised risk factors included aggression, receiving bad news, bereavement, domestic, family and financial stressors, low self-worth and recent negative experiences.^30,41,47,67^

#### Subtheme 2.2: perceived reasons for self-harm

The most frequently reported reason for self-harm by multiple staff roles was to ‘manipulate’ others.^9,38–43,45,48,49,52,62–64,67^ Prison staff listed benefits that ‘manipulative’ self-harm aimed to achieve, including more relaxed prison regimes; access to goods, medications, care or services; transfer to hospital and escape from other inmates.^41,42,45,48,49,63,67^ In contrast, some staff felt that self-harm was an expression of needs and/or a ‘cry for help’.^30,42,43,45,48,49,64,67^ Other commonly perceived functions of self-harm included ‘attention-seeking’,^30,41,42,44,45,48,62,64,67^ emotional expression and relieving tension,^30,40–42,44,47,63,67^ and a coping mechanism for difficult experiences.^40–42,47,63,67^

‘ … Cutting yourself and seeing the blood oozing out is a much more visual representation of a relief of tension than talking to somebody’ (Healthcare staff).^40^

Some staff described ‘copycat’ self-harm where inmates replicated that of others, ^40,41,48,62,63,67^ and reported prisoners being encouraged to harm themselves by peers and ‘less-seasoned staff’.^9^^,63,64^ Other described functions of self-harm included providing empowerment or control,^40,42,49,62,67^ giving enjoyment including sexual pleasure,^30,42,63,67^ and punishing oneself.^42,67^

#### Subtheme 2.3: relationships between self-harm and suicide

Non-fatal self-harm was often seen as distinct from and unrelated to suicide.^41,42,44^ Prison staff acknowledged that self-harm could result in death, but felt this was a result of ‘determined misadventure’ or going ‘too far’ and not a ‘genuine suicide attempt’.^41,42,64^ Some prison staff believed that suicide was unpreventable, stating that ‘if someone's determined to kill themselves, they'll always find a way’.^64^ It was frequently suggested that prisoners who intend to die use more lethal self-harm methods and conceal their intentions and distress more than those with non-suicidal motives.^41,42,44^

‘He set fire to himself in the exercise yard, he'd hung himself, he'd scratched his wrists, but he's more of a nuisance than an active suicide risk, but on this occasion, he went too far’ (Unspecified prison staff).^64^

### Theme 3: factors affecting staff attitudes toward self-harm

#### Subtheme 3.1: effects of repetitive self-harm

Repeated self-harm was viewed most negatively by prison staff, and often regarded as being fundamentally different from isolated incidents.^42–45,48,64^ Repetitive self-harm was described as ‘draining’ staff patience, optimism and resources, and was associated with critical comments, perceived ‘attention-seeking’ and staff frustration.^42,44,45,64^ Although some staff identified that ‘ongoing’ interactions with prisoners who repeatedly self-harm made them feel closer to them, many officers and healthcare staff reported that this was desensitising and reduced their empathy.^42,45^

‘So when he does it, any gesture, it's very hard to go oh, my god, it's really so so wrong and (.) poor thing kind of [ … ] when you sort of, for the seventh time gone taken him to hospital, treated the wound, and (.) I think it just set this relation can go a bit stale’ (Healthcare staff).^42^

#### Subtheme 3.2: effects of gender

Male staff more frequently recommended maintaining emotional distance from prisoners, whereas females emphasised relationship building.^67^

Male prisoners were thought to self-harm more severely and discreetly, generally afforded less sympathy and deemed to have less ‘genuine’, ‘serious’ or ‘complex’ issues relative to females.^42,63^ Prison staff occasionally portrayed self-harm as childish and emasculating, commenting that it was ‘such a young female kind of thing to do’.^42,64^

#### Subtheme 3.3: effects of job role

Healthcare staff generally reported a greater understanding of self-harm compared with prison officers. They more often identified situational risk factors within prison environments,^40,48^ commented on the effects of childhood trauma and abuse,^9^ and recognised self-harm as a coping mechanism for emotional distress.^42^ They also generally reported feeling more able to prevent self-harm.^41^ Compared with prison officers, healthcare staff less frequently delineated perceived ‘genuine’ and ‘non-genuine’ self-harm,^40,48^ and less often directly described self-harm as ‘manipulative’ or being enacted to punish staff.^42,45^

Compared with prison officers and healthcare staff, staff in specialist roles, such as governor, chaplaincy and suicide prevention coordinator, demonstrated more concern for the management of repetitive self-harm, more frequently emphasised its underlying causes and more frequently conceptualised people who self-harm as victims.^42^

One study found no variation in levels of expressed emotion toward self-harm between staff roles.^44^

### Theme 4: the effects of prison staff's experiences, perceptions and attitudes on self-harm management and prisoner interactions

#### Subtheme 4.1: labelling and dichotomising self-harm

Prison staff frequently dichotomised self-harm as being either ‘genuine/real’ or ‘non-genuine’ based upon their perceived functions of the behaviour. ‘Manipulation’ and ‘attention-seeking’ were typically labelled as ‘non-genuine’ illegitimate reasons for self-injury,^40–43,45,48,64^ and less severe ‘superficial’ self-harm injuries were more likely to be perceived as ‘non-genuine’.^48^

‘They are just attention seekers, they are taking away from the real problem, people who have real problems … ’ (Officer).^42^

Self-harm that was labelled ‘non-genuine’ or ‘manipulative’ was often regarded as less deserving of care and intervention, and some prison staff reported resisting opening ACCTs (Assessment, Care in Custody and Teamwork processes used in England and Wales to assess and manage self-harm risk) on these prisoners.^40,41,45,48,64^ Staff also described ‘manipulative’ and ‘non-genuine’ self-harm as requiring more punitive management, such as isolation, boundary setting and reduced staff attention.^9,64^ This contrasted to staff descriptions of optimal self-harm management, which generally involved building strong staff–prisoner relationships, getting to know individual prisoners and an emphasis on communication, rapport building and trust.^9,41,49,51,64,67^

‘The ones that I feel genuinely do have real problems and a genuine self-harm issue. I don't mind spending time with people who genuinely need help. I can't be doing with timewasters … ’ (Officer).^40^

Staff in one study recognised that patients whose self-harm is ‘non-genuine’ may also be struggling and require support.^48^

#### Subtheme 4.2: staff protecting themselves from the effects of prisoner self-harm

Staff commonly reported ‘building up tolerance’, and becoming ‘desensitised’ or ‘hardened’ to self-harm following repeated exposure.^39,41–43,49,52,64^ They additionally described maintaining emotional distance and becoming emotionally ‘switch[ed] off from prisoners’.^39,62,63,67^ These were generally construed as effective defence mechanisms to limit the emotional effects of self-harm and reduce burnout among prison staff. However, they were also thought to be associated with poorer risk identification and management, and intolerance, anger and cynicism toward prisoners.^41,42^

‘ … It would not affect me one way or another who or how many die through self-harm’ (Healthcare staff).^64^

Fear of being blamed for prisoners’ self-harm promoted defensive practices among prison staff. Staff described avoiding involvement in self-harm incidents, such as by avoiding night shifts and/or contact with prisoners who frequently self-harm.^42,43,50^ Staff also reported refraining from using personal discretion and adopting risk-averse practice to protect themselves.^41,43,45,52,64^ There were tensions described between healthcare and prison staff, whereby prison officers were reluctant to accept responsibility for caring for people who self-harm, particularly repetitive self-harm, and attributed this role to healthcare professionals,^42,45,52,64^ whereas healthcare staff felt that officers should assume greater ownership.^42,45^

‘They could stop self-harming becoming medicalised, by giving some sort of responsibility back to your discipline officers. All decisions seemed to be made by healthcare, which should really not be the case’ (Nurse).^45^

#### Subtheme 4.3: staff adherence to self-harm policies and procedures

To reduce intense workload and circumvent a lack of resources, staff described deviating from recognised self-harm policies and procedures. For example, although some staff reported having low thresholds to commence ACCT procedures and following them by the letter,^41,45,48,52^ others were discouraged by their colleagues from opening ACCTs,^62^ resisted doing so and/or waited for others to accept this responsibility.^41^ This was because of the perceived strain on staff time of completing multiple paperwork, which was felt to subtract from meaningful engagement with prisoners.^39,41,51,67^ Similarly, some staff recognised the importance of individualising self-harm management, whereas others treated self-harm processes as ‘tick-box’ tools.^39,41,42,49,67^ Prison officer attitudes and trust/distrust of specialist staff roles influenced the availability and effectiveness of multidisciplinary support for prisoners at risk of self-harm; for example, affecting the extent of specialist input during risk assessment and case reviews.^64^

### Quantitative data synthesis

Ten studies used quantitative methods alone,^30,53–61^ and six mixed methods.^62–67^ All 16 studies used surveys to obtain quantitative data relevant to this review.

#### Trauma Symptom Inventory

Two studies used the Trauma Symptom Inventory^,68^ tool to examine self-reported post-traumatic stress disorder (PTSD) symptoms among UK prison staff.^54,61^ Cassidy and Bruce found that 31.8% of prison staff closely involved in prisoner suicide in the past year reported clinical-level symptoms of PTSD.^54^ Furthermore, Wright et al discovered PTSD symptoms reported by 36.7% of prison officers, and that experience of prisoner suicide predicted such symptoms.^61^

#### Attitudes towards Prisoners who Self-Harm Scale

Two UK studies administered the Attitudes towards Prisoners who Self-Harm Scale (APSH) to prison staff, one in two high security prisons and one to prison officers attending prison service college training.^55,56^ This self-report scale measures attitudes relating to self-harm.^55^ In both studies, staff achieved similar mean scores, both indicating generally positive attitudes, but some negative attitudes present.^55,56^ Among prison officers attending Prison Service College, positive attitudes toward the treatment of prisoners, as measured by the General Attitudes towards Prisoners Scale (ATP), significantly predicted more favourable attitudes toward self-harm.^56^ Female staff scored statistically significantly higher on the APSH than males in the same study, indicating more positive attitudes.^56^ Staff's perceptions of prisoner's behavioural characteristics significantly influenced attitudes toward self-harm, with less positive attitudes toward ‘disruptive’ compared with ‘well-behaved’ prisoners.^56^

Another study administered the APSH and ATP to 176 prison officers in three Portuguese prisons.^59^ High proportions of prison staff believed that self-harm served ‘attention-seeking’ and ‘manipulative’ purposes and that prisoners who self-harm will not commit suicide. The majority of prison officers recognised self-harm as a coping mechanism. Perceptions of self-harm being ‘manipulative’ were more common among female officers, whereas male officers were significantly more likely to relate prisoner's self-harm to previous experiences of abuse. There was a significant positive correlation between prison officer's abilities to understand prisoners’ feelings and their understanding of self-harm. Officers that endorsed the strict discipline of prisoners were more likely to believe that prisoners who self-harm should be harshly treated and/or ignored, although this view was not shared by the majority.

#### Studies using statistical methods to analyse prison staff's attitudes, perceptions or experiences of self-harm

Five studies used quantitative methods to analyse factors affecting prison staff's experiences or perceptions of, or attitudes toward, self-harm.^30,53,57,60,64^

Slade and Lopresti utilised linear regression to examine factors associated with staff–prisoner relationships and staff resilience among 281 multidisciplinary UK prison staff and 169 community controls.^57^ They found that 61.2% of staff witnessed self-harm serious enough to warrant medical attention on ten or more occasions, which was associated with significantly less friendliness, understanding and support between staff and prisoners. More accepting attitudes toward suicide were predictive of better staff–prisoner relationships, along with staff members’ use of ‘surface acting’ and ‘deep acting’ emotional labour. Surface acting refers to the suppression of true feelings and/or presenting emotions that are different from those being felt, e.g. faking sadness and empathy. Deep acting, on the other hand, involves attempting to feel emotions that are deemed most appropriate, such as attempting to experience genuine empathy, rather than superficially faking this. Greater staff resilience was predicted by greater experience of prisoner suicide, advanced suicide prevention training, working in male prisons with low suicide and high self-harm rates, and greater deep acting and less surface acting of emotions. Staff in women's prisons perceived suicide as more preventable, displayed less condemning attitudes and reported less ‘faking of emotions’ compared with staff in men's prisons.

Wood-Schultz examined the effects of personal attitudes on the quality of suicide prevention responses by 84 prison officers, mental health and medical staff from the American Correctional Association.^60^ Positive attitudes toward prisoners were significantly associated with increased quality of suicide prevention responses among all staff, whereas attitudes toward death and suicide were not predictors.

A study of 76 correctional officers in Australia presented staff with vignettes depicting self-harm and asked them to rate causes and functions of the behaviour.^30^ Officers frequently linked self-harm to poor coping, depression and drug misuse. Other highly reported functions of self-harm by officers included ‘crying for help’, attention-seeking and releasing emotions, whereas suicidal functions were least frequently recognised. In contrast with evidence from qualitative studies,^42–45,48,64^ the severity and repetitiveness of self-harm did not significantly alter staff perceptions of its cause and function; however, low severity non-repetitive self-harm was linked by staff to prisoner distress, and higher severity self-harm was more often perceived as suicidal.^30^

A study of 1877 security staff in the USA investigated staff experiences and views of mental illness and healthcare in prisons, using clinical vignettes and self-administered surveys.^53^ Male and minority ethnic staff were significantly more likely to view prisoners, depicted in clinical vignettes, as at risk of self-harm. Prisoners were also more likely to be deemed at risk of self-harm if officers perceived their problems as caused by mental illness, chemical imbalance or genetics instead of ‘bad character’.

Staff and prisoner surveys and interviews, and participant observation, were used in UK prisons to evaluate the implementation and effectiveness of a programme to reduce self-harm and suicide.^64^ Multiple staff across several prisons reported finding self-harm management ‘extremely stressful’, and many strongly agreed that further training and support were required. Improved quality of life of prison staff was significantly associated with greater effectiveness of suicide prevention and suicide prevention effectiveness correlated highly with strong communication, good work culture, appropriate staff roles and responsibilities, and positive working relationships with managers. In prisons where many staff perceived self-harm as manipulative and threatening their authority, higher levels of prisoner distress existed.

#### Studies reporting descriptive statistics

Six studies reported the proportion of prison staff describing particular experiences, perceptions and attitudes relating to self-harm.^58,62,63,65–67^

Four papers used data collected from the same sample of 68 multidisciplinary prison staff in the UK, using questionnaires.^62,65–67^ The most commonly perceived reasons for prisoner self-harm included emotional expression, exerting control and gaining attention;^62,67^ 75% of prison staff thought that self-harm served to ‘manipulate’ others.^62,67^ Mixed views were expressed regarding staff's perceived knowledge of self-harm, with this being presented as both a strength and a challenge;^65–67^ 43% of prison staff reported needing more self-harm training, and lack of time was reported by 70% of staff as limiting optimal care.^65–67^

In a survey of 54 mental health professionals across 14 secure facilities in the USA, 91% of those working in prison viewed self-harm as a means to seek special treatment or transfer of location,^63^ 85% of staff viewed self-harm as a stress-coping mechanism, and a minority attributed self-harm to suicidal motives or severe mental disorder. Only 4% of mental health professionals could not recall a self-harm incident in the previous 6 months.

In a survey of 230 mental health professionals across 473 USA correctional facilities,^58^ 98% of staff knew a prisoner who self-harms in prison. The most witnessed method was scratching/cutting with objects, which generated the most concern among staff.

### Quality assessment

Quality assessment found that seven studies were good quality, 15 were moderate quality and ten were poor quality (Supplementary Appendix 4). Common study limitations included small sample sizes, non-random sampling, use of non-validated self-report measures and lack of information regarding the delivery and structure of staff interviews. All studies were cross-sectional in design, which prevented assessment of changes in staff experiences, perceptions and attitudes over time, and the ability to make causal inferences on identified associations.

Interrater reliability scores for the quality assessment of qualitative, quantitative and mixed-methods studies in this review were 87.5%, 90% and 83.3%, respectively, which increased to 100% following consensus between independent reviewers.

## Discussion

This systematic review identified 32 papers from five countries that provided data to answer the research question, ‘What are the experiences, perceptions and attitudes of prison staff regarding adult prisoners who self-harm?’. Both qualitative and quantitative data showed that prison staff report frequent exposure to many types of self-harm, and have a broad range of both positive and negative attitudes and perceptions regarding this. Perceptions that self-harm is ‘manipulative’, ‘non-genuine’ or ‘attention-seeking’ were associated with descriptions of poorer quality care and hostile behaviour toward prisoners in several qualitative studies, particularly for repetitive self-harm. Similarly, quantitative data revealed associations between prison staff's attitudes and perceptions, and the quality of their suicide prevention responses, self-harm management strategies, staff–prisoner relationships and levels of prisoner distress. Staff demonstrating positive attitudes and good understanding of self-harm described greater empathy and supportive management of prisoners. Several staff in qualitative studies reported experiencing intense emotions and distress from self-harm incidents, and found it difficult to manage self-harm within prisons; these emotions influenced their reported interactions with prisoners and capacity to care. The effects of self-harm on prison staff were similarly recognised in quantitative data, where multiple prison staff expressed symptoms of PTSD.

Many of the attitudes and experiences described by prison staff have been demonstrated in other settings, e.g. among hospital workers. These include strong emotional reactions to self-harm, positive and negative staff attitudes and behaviours, feelings of uncertainty and inadequacy, and a perceived lack of time and resources to effectively care for self-harm.^69^ A specific challenge of self-harm management in prisons is the reported tensions between balancing security, justice and punishment with compassionate healthcare, which creates confusion and conflict among prison staff. This tension of ‘care versus custody’ is well-recognised in the forensic literature.^70^

It is important to consider how prison staff's perceptions and attitudes are perceived by prisoners. In prior research, prisoners who self-harm have described prison staff as ‘approachable’, helpful and providing strong emotional support.^47,65,66^ They have also highlighted the beneficial effects of positive relationships and conversations with prison staff to alleviate distress.^47,65,66^ In contrast, some studies have reported prisoner views that they are not adequately listened to or cared for following self-harm, and that prison staff lack understanding of this.^42,47,65,66^ Prisoners who self-harm have also reported fears of being labelled ‘attention seekers’, and beliefs that less dangerous self-harm receives less concern and support from staff.^65^ Perceptions of inadequate care and hostility from prison staff have been reported by prisoners to increase their risks of self-harm.^42,71^ Prison staff's descriptions of intense workloads and lack of resources are echoed in prisoner reports of finding prison staff ‘too busy’ and not ‘geared up’ for managing self-harm.^42^

It is notable that prison staff described feelings and emotions that might mirror those experienced by prisoners who self-harm, such as feeling ‘helpless’ and ‘frustrated’. Prison officers often requested mental health training, which might include helping them to understand their own reactions to self-harm, in turn potentially improving emotional well-being.

Many of the risk factors and reasons for self-harm identified by prison staff in this review are corroborated by previous research.^72–74^ Several of these risk factors are unique to prisons, such as isolation and boredom from excess time in cells and bullying and violence between prisoners. Prison staff's descriptions of inmates ‘copying’ self-harm are supported by ‘clustering’ of self-harm incidents in prisons across time and location.^2^ It is therefore suggested that self-harm management strategies extend beyond individuals, to other prisoners in the locality.^2,75^

Prison staff frequently underestimated the association between self-harm and suicide, as self-harm strongly predicts subsequent suicide, both in prison and following release.^76,77^ Furthermore, perceived ‘manipulative’ reasons for self-harm among prisoners can co-exist with suicidal intent and lethal behaviour,^78^ which is out of keeping with staff views that ‘manipulative’ self-harm requires less intervention. These misconceptions, particularly around the risk of subsequent suicide, demonstrate an important training need.

This review highlights the significant demands on prison staff that routinely face high rates and numerous types of prisoner self-harm. Staff often perceived themselves to lack training and skills to prevent and manage self-harm, and fear blame and feel unsupported by the prison system. According to the job demand–control–support model^79^ and job demands–resources model,^80^ this imbalance between high demands and perceived lack of control, support and resources can create a predisposition to occupational stress. Furthermore, witnessing self-harm and suicide can be traumatising in itself.^81^ This could partly explain the high rates of occupational stress and mental illness within the prison workforce.^82,83^ In the studies reviewed, many prison staff described coping by becoming emotionally distanced from prisoners and desensitised to self-harm; this might suggest compassion fatigue, whereby a person's ability to provide empathetic care declines following exposure to traumatic events.^84^ Improving prison officer support would likely not only improve staff well-being and retention, but also indirectly improve care for prisoners.

### Implications for clinical practice and service improvement

Reducing self-harm in prisons requires a multifaceted approach that addresses the individual needs of prisoners, the attitudes and perceptions of prison staff, and the prison environment. In accord with National Institute for Health and Care Excellence guidelines, all prison staff in contact with people who self-harm should receive appropriate training.^85^ This training should identify and challenge any negative attitudes and perceptions, and support staff in developing more positive attitudes through education and reflection. Caring attitudes and empathy should be reinforced through positive recognition, whereas labelling self-harm as ‘non-genuine’ and punitively treating prisoners risks their safety and should be actively discouraged. Many prison staff in this review felt that current training was insufficient, suggesting a need to involve staff in co-designing training and identifying their learning needs. Ensuring recognition of the strong association between self-harm and subsequent prisoner suicide should be an important aspect of staff training.

Prison services should also ensure effective policies for tackling bullying, boredom, isolation and substance misuse, all of which were identified to precipitate self-harm. Having sufficient resources, including adequate staffing and access to specialist mental health teams, is vital to prisoner safety. Risk management systems, such as ACCT processes, can improve the quality of care delivered to prisoners who self-harm;^86^ however, staff engagement with these processes varied in the present review, highlighting the roles of clinical governance and audit to monitor their effectiveness and address barriers to effective implementation.

Prison services should work collaboratively with staff to design and implement support structures that are accessible and acceptable to the workforce, and that are actively prescribed. Stress management and coping skills training for dealing with the emotional effects of self-harm could improve staff well-being and compassion. Good working relationships and role clarity are protective for prison officer well-being.^82^

### Strengths and limitations of the review

This review has several strengths. To our knowledge, this is the first systematic review and mixed-methods synthesis focussing on the experiences, perceptions and attitudes of prison staff working with prisoners who self-harm. The review included a large sample of many staff roles within prisons, and identified several facilitators and barriers to self-harm management and prevention, with clear implications for research and policy. Furthermore, the inclusion of both qualitative and quantitative data allowed a detailed understanding of the extent and nature of the review findings.

Limitations of this review include the high level of heterogeneity between studies, which precluded meta-analysis. The frequent use of self-report measures and non-validated questionnaires to measure staff's attitudes and perceptions meant that several studies were of low quality. The generalisability of the review findings is limited by the exclusion of non-English language articles and underrepresentation of studies conducted outside of the UK. Excluding studies conducted in YOIs was important to ensure that staff attitudes, perceptions and experiences regarding self-harm related to adult, rather than adolescent, prisoners; however, this means that some information regarding young adults who self-harm may have been excluded from the review. All included studies were conducted before the COVID-19 pandemic, which has significantly affected self-harm within prisons, as well as prison environments, staffing levels and staff well-being.^6,87–89^

### Future research

Reviewing the perspective of prisoners would be useful to allow a detailed understanding of how staff attitudes are perceived, and might affect self-harm. Future research is also needed to unpick potential associations between rates of self-harm in prisons and the attitudes, perceptions and well-being of prison staff. The overall quality of research in this area would be strengthened by larger sample sizes and validated measures for assessing prison staff attitudes and beliefs about self-harm. Longitudinal studies are also needed to assess how staff attitudes and behaviours are developed over time. Future research should additionally investigate the effects of COVID-19 on self-harm in prisons. Data from England and Wales indicate that rates of self-harm have increased for female prisoners but decreased for male prisoners during the pandemic.^6^

## Data Availability

Data availability is not applicable to this article as no new data were created or analysed in this study.

## Supplementary material

For supplementary material accompanying this paper visit http://doi.org/10.1192/bjo.2022.70. click here to view supplementary material

## References

[1] Ministry of Justice. Guide to Safety in Custody Statistics. Ministry of Justice, 2010 (https://assets.publishing.service.gov.uk/government/uploads/system/uploads/attachment_data/file/676151/safety-in-custody-statistics-guide.pdf).

[2] Hawton K, Linsell L, Adeniji T, Sariaslan A, Fazel S. Self-harm in prisons in England and Wales: an epidemiological study of prevalence, risk factors, clustering and subsequent suicide. Lancet Psychiatry 2014; 383(9923): 1147–54. 10.1016/S0140-6736(13)62118-2 PMC3978651 24351319

[3] Fazel S, Ramesh T, Hawton K. Suicide in prisons: an international study of prevalence and contributory factors. Lancet Psychiatry 2017; 4(12): 946–52. 29179937 10.1016/S2215-0366(17)30430-3 PMC6066090

[4] Fazel S, Benning R. Suicides in female prisoners in England and Wales, 1978–2004. Br J Psychiatry 2009; 194(2): 183–4. 19182185 10.1192/bjp.bp.107.046490

[5] Fazel S, Benning R, Danesh J. Suicides in male prisoners in England and Wales, 1978–2003. Lancet 2005; 366(9493): 1301–2. 16214601 10.1016/S0140-6736(05)67325-4

[6] Her Majesty's Prison & Probation Service, Ministry of Justice. *Safety in Custody Statistics, England and Wales: Deaths in Prison Custody to December 2021, Assaults and Self-Harm to September 2021*. Ministry Of Justice, 2022 (https://www.gov.uk/government/statistics/safety-in-custody-quarterly-update-to-september-2021/safety-in-custody-statistics-england-and-wales-deaths-in-prison-custody-to-december-2021-assaults-and-self-harm-to-september-2021).

[7] Favril L, Laenen FV, Vandeviver C, Audenaert K. Suicidal ideation while incarcerated: prevalence and correlates in a large sample of prisoners in Flanders, Belgium. Int J Law Psychiatry 2017; 55: 19–28. 29157508 10.1016/j.ijlp.2017.10.005

[8] The Howard League for Penal Reform. The Cost of Prison Suicide: Research Briefing. The Howard League for Penal Reform, 2016 (https://howardleague.org/wp-content/uploads/2016/03/The-cost-of-prison-suicide.pdf).

[9] Smith HP, Power J, Usher AM, Sitren AH, Slade K. Working with prisoners who self-harm: a qualitative study on stress, denial of weakness, and encouraging resilience in a sample of correctional staff. Crim Behav Ment Health 2019; 29(1): 7–17. 30609122 10.1002/cbm.2103

[10] Herbert A, Gilbert R, Cottrell D, Li L. Causes of death up to 10 years after admissions to hospitals for self-inflicted, drug-related or alcohol-related, or violent injury during adolescence: a retrospective, nationwide, cohort study. Lancet 2017; 390(10094): 577–87. 28552365 10.1016/S0140-6736(17)31045-0

[11] Bergen H, Hawton K, Waters K, Ness J, Cooper J, Steeg S, Premature death after self-harm: a multi-centre cohort study. Lancet 2012; 380(9853): 1568–74. 22995670 10.1016/S0140-6736(12)61141-6

[12] UK Government, Department of Health. Preventing Suicide in England: A Cross-Government Outcomes Strategy to Save Lives. The Stationery Office, 2012 (https://assets.publishing.service.gov.uk/government/uploads/system/uploads/attachment_data/file/430720/Preventing-Suicide-.pdf).

[13] HM Prison Service. Competency and Qualities Framework. HM Prison Service (https://www.justice.gov.uk/downloads/jobs/hmps-competence-framework.pdf).

[14] Clements C, Turnbull P, Hawton K, Geulayov G, Waters K, Ness J, Rates of self-harm presenting to general hospitals: a comparison of data from the multicentre study of self-harm in England and hospital episode statistics. BMJ Open 2016; 6: e009749. 10.1136/bmjopen-2015-009749 PMC4762081 26883238

[15] Friedman T, Newton C, Coggan C, Hooley S, Patel R, Rickard M, Predictors of A + E staff attitudes to self-harm patients who use self-laceration: influence of previous training and experience. J Psychosom Res 2006; 60(3): 273–7. 16516659 10.1016/j.jpsychores.2005.07.007

[16] Anderson M, Standen P, Noon J. Nurses’ and doctors’ perceptions of young people who engage in suicidal behaviour: a contemporary grounded theory analysis. Int J Nurs Stud 2003; 40(6): 587–97. 12834924 10.1016/s0020-7489(03)00054-3

[17] Hopkins C. ‘But what about the really ill, poorly people?’ (An ethnographic study into what it means to nurses on medical admissions units to have people who have harmed themselves as their patients). J Psychiatr Ment Health Nurs 2002; 9(2): 147–54. 11966983 10.1046/j.1365-2850.2002.00473.x

[18] Anderson M, Standen P, Nazir S, Noon J. Nurses’ and doctors’ attitudes towards suicidal behaviour in young people. Int J Nurs Stud 2000; 37(1): 1–11. 10687805 10.1016/s0020-7489(99)00057-7

[19] Creed FH, Pfeffer JM. Attitudes of house-physicians towards self-poisoning patients. Med Educ 1981; 15(5): 340–5. 7266400 10.1111/j.1365-2923.1981.tb02500.x

[20] Shaw DG, Sandy PT. Mental health nurses’ attitudes toward self-harm: curricular implications. Health SA Gesondheid 2016; 21: 406–14.

[21] Thompson AR, Powis J, Carradice A. Community psychiatric nurses’ experience of working with people who engage in deliberate self-harm. Int J Ment Health Nurs 2008; 17(3): 153–61. 18460076 10.1111/j.1447-0349.2008.00533.x

[22] Saunders KEA, Hawton K, Fortune S, Farrell S. Attitudes and knowledge of clinical staff regarding people who self-harm: a systematic review. J Affect Disord 2012; 139(3): 205–16. 21925740 10.1016/j.jad.2011.08.024

[23] Patterson P, Whittington R, Bogg J. Measuring nurse attitudes towards deliberate self-harm: the Self-harm Antipathy Scale (SAS). J Psychiatr Ment Health Nurs 2007; 14(5): 438–45. 17635251 10.1111/j.1365-2850.2007.01102.x

[24] Smith S. Perceptions of service provision for clients who self-injure in the absence of expressed suicidal intent. J Psychiatr Ment Health Nurs 2002; 9(5): 595–601. 12358713 10.1046/j.1365-2850.2002.00512.x

[25] Rayner GC, Allen SL, Johnson M. Countertransference and self injury: a cognitive behavioural cycle. J Adv Nurs 2005; 50(1): 12–9. 15788061 10.1111/j.1365-2648.2005.03344.x

[26] Pompili M, Girardi P, Ruberto A, Kotzalidis GD, Tatarelli R. Emergency staff reactions to suicidal and self-harming patients. Eur J Emerg Med 2005; 12(4): 169–78. 16034262 10.1097/00063110-200508000-00005

[27] Leach MJ. Rapport: a key to treatment success. Complement Ther Clin Pract 2005; 11(4): 262–5. 16290897 10.1016/j.ctcp.2005.05.005

[28] Wiklander M, Samuelsson M, Asberg M. Shame reactions after suicide attempt. Scand J Caring Sci 2003; 17(3): 293–300. 12919465 10.1046/j.1471-6712.2003.00227.x

[29] Patterson P, Whittington R, Bogg J. Testing the effectiveness of an educational intervention aimed at changing attitudes to self-harm. J Psychiatr Ment Health Nurs 2007; 14(1): 100–5. 17244012 10.1111/j.1365-2850.2007.01052.x

[30] Pannell J, Howells K, Day A. Prison officer's beliefs regarding self-harm in prisoners: an empirical investigation. Int J Forensic Psychol 2003; 1(1): 103–10.

[31] Cleaver K, Meerabeau L, Maras P. Attitudes towards young people who self-harm: age, an influencing factor. J Adv Nurs 2014; 70(12): 2884–96. 24862740 10.1111/jan.12451

[32] Critical Appraisals Skills Programme (CASP) UK. *CASP Checklists*. CASP UK, 2018 (https://casp-uk.net/casp-tools-checklists).

[33] National Heart, Lung, and Blood Institute (NHLBI). Study Quality Assessment Tools. US Department of Health and Human Services, 2021 (https://www.nhlbi.nih.gov/health-topics/study-quality-assessment-tools).

[34] Hong QN, Pluye P, Fàbregues S, Bartlett G, Boardman F, Cargo M, *Mixed Methods Appraisal Tool (MMAT) Version 2018*. McGill University, 2018 (http://mixedmethodsappraisaltoolpublic.pbworks.com/w/file/fetch/127916259/MMAT_2018_criteria-manual_2018-08-01_ENG.pdf).

[35] QSR International Pty Ltd. *NVivo for Windows (Version 12)*. QSR International Pty Ltd, 2018 (https://www.qsrinternational.com/nvivo-qualitative-data-analysis-software/home).

[36] Thomas J, Harden A. Methods for the thematic synthesis of qualitative research in systematic reviews. BMC Med Res Methodol 2008; 8: 45. 18616818 10.1186/1471-2288-8-45 PMC2478656

[37] Petticrew M, Roberts H. Synthesising the evidence. In: Systematic Reviews in the Social Sciences (eds M Petticrew, H Roberts): 164–214. Blackwell Publishing Ltd, 2006.

[38] Bantjes J, Swartz L, Niewoudt P. Human rights and mental health in post-apartheid South Africa: lessons from health care professionals working with suicidal inmates in the prison system. BMC Int Health Hum Rights 2017; 17(1): 29. 29025417 10.1186/s12914-017-0136-0 PMC5639765

[39] The Howard League for Penal Reform and Centre for Mental Health. Preventing Prison Suicide: Staff Perspectives. The Howard League for Penal Reform, 2017 (https://howardleague.org/publications/preventing-prison-suicide-staff-perspectives/).

[40] Kenning C, Cooper J, Short V, Shaw J, Abel K, Chew-Graham C. Prison staff and women prisoner's views on self-harm; their implications for service delivery and development: a qualitative study. Crim Behav Ment Health 2010; 20: 274–84. 20603816 10.1002/cbm.777

[41] Ludlow A, Schmidt B, Akoensi T, Liebling A, Giacomantonio C, Sutherland A. Self-Inflicted Deaths in NOMS’ Custody amongst 18–24 Year Olds. RAND Corporation, 2015 (https://static1.squarespace.com/static/5c5ae65ed86cc93b6c1e19a3/t/5ee9d9c86fa0cb590b90b4b5/1592383950684/Self-Inflicted-Deaths-in-NOMS%E2%80%99-Custody-amongst-18%E2%80%9324-Year-Olds-Staff-Experience-Knowledge-and-Views.pdf).

[42] Marzano L. *Self-harm in a men's prison: staff's and prisoners’ perspectives*. *D Phil doctoral thesis* Department of Psychology, Middlesex University, 2007.

[43] Marzano L, Adler JR, Ciclitira K. Responding to repetitive non-suicidal self-harm in an English male prison: Staff experiences, reactions, and concerns. Leg Crim Psychol 2013; 20(2): 241–54.

[44] Moore E, Andargachew S, Taylor P. Working with women prisoners who seriously harm themselves: ratings of staff expressed emotion (EE). Crim Behav Ment Health 2010; 21(1): 63–74. 21259370 10.1002/cbm.795

[45] Ramluggun P. A critical exploration of the management of self-harm in a male custodial setting: qualitative findings of a comparative analysis of prison staff views on self-harm. J Forensic Nurs 2013; 9(1): 23–34. 24158098 10.1097/JFN.0b013e31827a5984

[46] Rivlin A. Self-harm and suicide at Grendon Therapeutic Community Prison. Prison Serv J 2007; 173: 34–38.

[47] Rivlin A. Suicide and self-injurious behaviours at HMP Grendon. In Grendon and the Emergence of Forensic Therapeutic Communities: Developments in Research and Practice (eds R Shuker, E Sullivan): 265–280. John Wiley & Sons Ltd, 2010.

[48] Short V, Cooper J, Shaw J, Kenning C, Abel K, Chew-Graham C. Custody vs care: attitudes of prison staff to self-harm in women prisoners – a qualitative study. J Forensic Psychiatry Psychol 2009; 20(3): 408–26.

[49] Sweeney F, Clarbour J, Oliver A. Prison officers’ experiences of working with adult male offenders who engage in suicide-related behaviour. J Forensic Psychiatry Psychol 2018; 29(3): 467–82.

[50] Tait S. A typology of prison officer approaches to care. Eur J Criminol 2011; 8(6): 440–54.

[51] Walker T, Shaw J, Hamilton L, Turpin C, Reid C, Abel K. Supporting imprisoned women who self-harm: exploring prison staff strategies. J Crim Psychol 2016; 6(4): 173–86.

[52] Walker T, Shaw J, Hamilton L, Turpin C, Reid C, Abel K. ‘Coping with the job’: prison staff responding to self-harm in three English female prisons: a qualitative study. J Forensic Psychiatry Psychol 2017; 28(6): 811–24.

[53] Callahan L. Correctional officer attitudes toward inmates with mental disorders. Int J Forensic Ment Health 2004; 3(1): 37–54.

[54] Cassidy T, Bruce S. Dealing with death in custody: psychosocial consequences for correctional staff. J Correct Health Care 2019; 25(4): 304–12. 31736409 10.1177/1078345819879752

[55] Garbutt K, Casey H. Attitudes towards prisoners who self harm scale: a psychometric evaluation. J Aggress Confl Peace Res 2015; 7(3): 158–66.

[56] Ireland JL, Quinn K. Officer attitudes towards adult male prisoners who self-harm: development of an attitudinal measure and investigation of sex differences. Aggress Behav 2007; 33(1): 63–72. 17441007 10.1002/ab.20168

[57] Slade K, Lopresti S. Research Findings: Promoting Resilience in Prison Staff. Nottingham Trent University, 2013 (http://irep.ntu.ac.uk/id/eprint/2343).

[58] Smith HP, Kaminski RJ. Self-injurious behaviors in state prisons: findings from a national survey. Crim Justice Behav 2010; 38(1): 26–41.

[59] Sousa M, Gonçalves RA, Cruz AR, de Castro Rodrigues A. Prison officers’ attitudes towards self-harm in prisoners. Int J Law Psychiatry 2019; 66: 101490. 31706411 10.1016/j.ijlp.2019.101490

[60] Wood-Schultz T. Variability in personal characteristics among professional staff providing suicide prevention responses in correctional settings. Doctoral thesis, Capella University, 2012.

[61] Wright L, Borrill J, Teers R, Cassidy T. The mental health consequences of dealing with self-inflicted death in custody. Couns Psychol Q 2006; 19(2): 165–80.

[62] Cresswell M, Karimova Z, Ward J. Women, self-harm, and the moral code of the prison. Ethical Hum Psychol Psychiatry 2018; 20(1): 27–42.

[63] DeHart DD, Smith HP, Kaminski RJ. Institutional responses to self-injurious behaviour among inmates. J Correct Healthcare 2009; 15(2): 129–41. 10.1177/1078345809331444 19477817

[64] Liebling A, Tait S, Durie L, Stiles A, Harvey J. An Evaluation of the Safer Locals Programme. Cambridge Institute of Criminology Prisons Research Centre, 2005 (https://citeseerx.ist.psu.edu/viewdoc/download?doi=10.1.1.369.3964&rep=rep1&type=pdf).

[65] Ward J, Bailey D. At arms length: the development of a self-injury training package for prison staff through service user involvement. J Ment Health Train Educ Pract 2011; 6(4): 175–85.

[66] Ward J, Bailey D. A participatory action research methodology in the management of self-harm in prison. J Ment Health 2013; 22(4): 306–16. 23323726 10.3109/09638237.2012.734645

[67] Ward CJ. Women's imprisonment, self-harm and emancipatory research: Developing a framework for transformative research in a women's prison. Doctoral thesis, School of Applied Social Science, Durham University, 2014.

[68] Briere J, Elliot DM, Harris K, Cotman A. Trauma symptom inventory: psychometrics and association with childhood and adult victimization in clinical samples. J Interpers Violence 1995; 10(4): 387–401.

[69] O'Connor S, Glover L. Hospital staff experiences of their relationships with adults who self-harm: a meta synthesis. Psychol Psychother 2017; 90(3): 480–501. 28035740 10.1111/papt.12113

[70] Mills A, Kendall K. Care versus custody: challenges in the provision of prison mental health care. In Mental Health in Prisons. Palgrave Studies in Prisons and Penology (eds A Mills, K Kendall): 105–29. Palgrave Macmillan, 2018.

[71] Ciclitira K, Adler J. The impact of prison staff responses on self-harming behaviours: prisoner's perspectives. Br J Clin Psychol 2012; 51(1): 4–18. 22268538 10.1111/j.2044-8260.2010.02007.x

[72] Power J, Brown SL, Usher AM. Non-suicidal self-injury in women offenders: motivations, emotions, and precipitating evens. Int J Forensic Ment Health 2013; 12(2013): 192–204.

[73] Snow L. Prisoners’ motives for self-injury and attempted suicide. Br J Forensic Pract 2002; 4(4): 18–29.

[74] Favril L, Yu R, Hawton K, Fazel S. Risk factors for self-harm in prison: a systematic review and meta-analysis. Lancet Psychiatry 2020; 7(8): 682–91. 32711709 10.1016/S2215-0366(20)30190-5 PMC7606912

[75] Pope L. Self-Harm by Adult Men in Prison: A Rapid Evidence Assessment. HM Prison and Probation Service, 2018 (https://www.gov.uk/government/publications/self-harm-by-adult-men-in-prison-a-rapid-evidence-assessment).

[76] Humber N, Webb R, Piper M, Appleby L, Shaw J. A national case-control study of risk factors among prisoners in England and Wales. Soc Psychiatry Psychiatr Epidemiol 2013; 48(7): 1177–85. 23232691 10.1007/s00127-012-0632-4

[77] Fazel S, Cartwright J, Norman-Nott A, Hawton K. Suicide in prisoners: a systematic review of risk factors. J Clin Psychiatry 2008; 69(11): 1721–31. 19026254

[78] Dear GE, Thomson DM, Hills AM. Self-harm in prison: manipulators can also be suicide attempters. Crim Justice Behav 2000; 27(2): 160–75.

[79] Karasek R, Theorell R. Healthy Work: Stress, Productivity, and the Reconstruction of Working Life. Basic Books, 1990.

[80] Demerouti E, Bakker AB, Nachreiner F, Schaufeli WB. The job demands-resources model of burnout. J Appl Psychol 2001; 86(3): 499–512. 11419809

[81] Bell S, Hopkin G, Forrester A. Exposure to traumatic events and experience of burnout, compassion fatigue and compassion satisfaction among prison mental health staff: an exploratory survey. Issues Ment Health Nurs 2019; 40(4): 304–9. 30742547 10.1080/01612840.2018.1534911

[82] Kinman G, Clements AJ, Hart J. Job demands, resources and mental health in UK prison officers. Occup Med 2017; 67(6): 456–60. 10.1093/occmed/kqx091 28898963

[83] Finney C, Stergiopoulos E, Hensel J, Bonato S, Dewa CS. Organizational stressors associated with job stress and burnout in correctional officers: a systematic review. BMC Public Health 2013; 13: 82. 23356379 10.1186/1471-2458-13-82 PMC3564928

[84] Sinclair S, Raffin-Bouchal S, Venturato L, Mijovic-Kondejewski J, Smith-MacDonald L. Compassion fatigue: a meta-narrative review of the healthcare literature. Int J Nurs Stud 2017; 69: 9–24. 28119163 10.1016/j.ijnurstu.2017.01.003

[85] National Institute for Health and Care Excellence (NICE). Self-Harm in Over 8s: Short-Term Management and Prevention of Recurrence. *Clinical Guideline [CG16].* NICE, 2004 (https://www.nice.org.uk/guidance/cg16/chapter/1-Guidance#support-and-advice-for-people-who-repeatedly-self-harm). 31891461

[86] Humber N, Hayes A, Senior J, Fahy T, Shaw J. Identifying, monitoring and managing prisoners at risk of self-harm/suicide in England and Wales. J Forensic Psychiatry Psychol 2011; 22(1): 22–51.

[87] Hewson T, Green R, Shepherd A, Hard J, Shaw J. The effects of COVID-19 on self-harm in UK prisons. BJPsych Bull 2021; 45(3): 131–3. 32669158 10.1192/bjb.2020.83 PMC7411440

[88] Hewson T, Shepherd A, Hard J, Shaw J. Effects of the COVID-19 pandemic on the mental health of prisoners. Lancet Psychiatry 2020; 7(7): 568–70. 32563298 10.1016/S2215-0366(20)30241-8 PMC7302764

[89] Johnson L, Gutridge K, Parkes J, Roy A, Plugge E. Scoping review of mental health in prisons through the COVID-19 pandemic. BMJ Open; 11(5): e046547. 10.1136/bmjopen-2020-046547 PMC8727680 33986064

