# Supplementary material for: A systematic review and mixed-methods synthesis of the experiences, perceptions and attitudes of prison staff regarding adult prisoners who self-harm
Source: BJPsych Open. 2022 Jun 6;8(4):e102. doi: 10.1192/bjo.2022.70 (PMC9230562; doi:10.1192/bjo.2022.70)
Supplement: Supplementary file 1 [file S2056472422000709sup001.docx]

**Supplementary Material**

**Appendix 1 – Database search strategy**

| **#** | **Database** | **Search term** | **Results** |
| --- | --- | --- | --- |
| 1 | Medline | (prison* OR inmate* OR offender* OR convict*).ti,ab | 31853 |
| 2 | Medline | PRISONERS/ | 16320 |
| 3 | Medline | CRIMINALS/ | 4762 |
| 4 | Medline | (1 OR 2 OR 3) | 40531 |
| 5 | Medline | (staff OR officer* OR governor* OR nurse* OR physician* OR employee* OR doctor*).ti,ab | 866249 |
| 6 | Medline | "MEDICAL STAFF"/ OR exp "NURSING STAFF"/ | 67771 |
| 7 | Medline | (5 OR 6) | 895132 |
| 8 | Medline | (self AND (wound* OR mutilat* OR harm* OR injury OR injuries)).ti,ab | 39328 |
| 9 | Medline | (auto mutilation OR automutilation).ti,ab | 144 |
| 10 | Medline | exp "SELF-INJURIOUS BEHAVIOR"/ | 69375 |
| 11 | Medline | (DSH OR deliberate self harm*).ti,ab | 1928 |
| 12 | Medline | (suicid*).ti,ab | 75091 |
| 13 | Medline | SUICIDE/ | 38629 |
| 14 | Medline | (cutting).ti,ab | 32765 |
| 15 | Medline | "SELF MUTILATION"/ | 3194 |
| 16 | Medline | (8 OR 9 OR 10 OR 11 OR 12 OR 13 OR 14 OR 15) | 162595 |
| 17 | Medline | (attitude* OR perception* OR experience* OR opinion* OR understand* OR perspective* OR view* OR feel*).ti,ab | 2999856 |
| 18 | Medline | ATTITUDE/ | 46737 |
| 19 | Medline | PERCEPTION/ | 34034 |
| 20 | Medline | COMPREHENSION/ | 14088 |
| 21 | Medline | (COMPREHENSION OR comprehend).ti,ab | 27958 |
| 22 | Medline | exp EMOTIONS/ | 235271 |
| 23 | Medline | exp "ATTITUDE OF HEALTH PERSONNEL"/ | 155820 |
| 24 | Medline | (17 OR 18 OR 19 OR 20 OR 21 OR 22 OR 23) | 3286827 |
| 25 | Medline | (4 AND 7 AND 16 AND 24) | 139 |
| 26 | Medline | 25 [DT 2000-2020] [Peer reviewed] [Languages English] | 29 |
| 27 | Medline | PRISONS/ | 9465 |
| 28 | Medline | (4 OR 27) | 43614 |
| 29 | Medline | (7 AND 16 AND 24 AND 28) | 141 |
| 30 | EMBASE | (prison* OR inmate* OR offender* OR convict*).ti,ab | 39161 |
| 31 | EMBASE | PRISONER/ OR PRISONERS/ OR PRISONS/ | 25959 |
| 32 | EMBASE | OFFENDER/ | 13299 |
| 33 | EMBASE | (30 OR 31 OR 32) | 55282 |
| 34 | EMBASE | (staff OR officer* OR governor* OR nurse* OR physician* OR employee* OR doctor*).ti,ab | 1150418 |
| 35 | EMBASE | "MEDICAL STAFF"/ | 35354 |
| 36 | EMBASE | "NURSING STAFF"/ | 68768 |
| 37 | EMBASE | (34 OR 35 OR 36) | 1189704 |
| 38 | EMBASE | (self AND (wound* OR mutilat* OR harm* OR injury OR injuries)).ti,ab | 54790 |
| 39 | EMBASE | (auto mutilation OR automutilation).ti,ab | 145 |
| 40 | EMBASE | AUTOMUTILATION/ | 17427 |
| 41 | EMBASE | (DSH OR deliberate self harm*).ti,ab | 2419 |
| 42 | EMBASE | SUICIDE/ OR "SUICIDE ATTEMPT"/ | 78496 |
| 43 | EMBASE | (cutting).ti,ab | 40800 |
| 44 | EMBASE | (38 OR 39 OR 40 OR 41 OR 42 OR 43) | 175317 |
| 45 | EMBASE | (attitude* OR perception* OR experience* OR opinion* OR understand* OR perspective* OR view* OR feel*).ti,ab | 3891783 |
| 46 | EMBASE | exp "HEALTH PERSONNEL ATTITUDE"/ | 180571 |
| 47 | EMBASE | ATTITUDE/ | 58771 |
| 48 | EMBASE | exp EMOTION/ | 568617 |
| 49 | EMBASE | COMPREHENSION/ | 29466 |
| 50 | EMBASE | (COMPREHENSION OR comprehend).ti,ab | 35126 |
| 51 | EMBASE | (45 OR 46 OR 47 OR 48 OR 49 OR 50) | 4400922 |
| 52 | EMBASE | (33 AND 37 AND 44 AND 51) | 176 |
| 53 | EMBASE | 52 [DT 2000-2020] [Priority journals] [Publication types Article OR Journal OR Review] [English language] | 28 |
| 54 | PsycINFO | (prison* OR inmate* OR offender* OR convict*).ti,ab | 60911 |
| 55 | PsycINFO | PRISONERS/ | 14166 |
| 56 | PsycINFO | exp "CRIMINAL OFFENDERS"/ | 19655 |
| 57 | PsycINFO | (54 OR 55 OR 56) | 69099 |
| 58 | PsycINFO | (staff OR officer* OR governor* OR nurse* OR physician* OR employee* OR doctor*).ti,ab | 289429 |
| 59 | PsycINFO | exp "MEDICAL PERSONNEL"/ | 80753 |
| 60 | PsycINFO | exp "HEALTH PERSONNEL"/ | 159571 |
| 61 | PsycINFO | exp NURSES/ | 30932 |
| 62 | PsycINFO | (58 OR 59 OR 60 OR 61) | 380400 |
| 63 | PsycINFO | (self AND (wound* OR mutilat* OR harm* OR injury OR injuries)).ti,ab | 25404 |
| 64 | PsycINFO | "SELF-INJURIOUS BEHAVIOR"/ OR "SELF-INFLICTED WOUNDS"/ OR "SELF-MUTILATION"/ OR SUICIDE/ | 38104 |
| 65 | PsycINFO | (auto mutilation OR automutilation).ti,ab | 83 |
| 66 | PsycINFO | (DSH OR deliberate self harm*).ti,ab | 1220 |
| 67 | PsycINFO | (suicid*).ti,ab | 62440 |
| 68 | PsycINFO | (cutting).ti,ab | 7279 |
| 69 | PsycINFO | (63 OR 64 OR 65 OR 66 OR 67 OR 68) | 92761 |
| 70 | PsycINFO | (attitude* OR perception* OR experience* OR opinion* OR understand* OR perspective* OR view* OR feel*).ti,ab | 1704275 |
| 71 | PsycINFO | exp "HEALTH PERSONNEL ATTITUDES"/ OR ATTITUDES/ | 49641 |
| 72 | PsycINFO | exp EMOTIONS/ | 346777 |
| 73 | PsycINFO | (COMPREHENSION OR comprehend).ti,ab | 46258 |
| 74 | PsycINFO | COMPREHENSION/ | 17534 |
| 75 | PsycINFO | (70 OR 71 OR 72 OR 73 OR 74) | 1932366 |
| 76 | PsycINFO | (57 AND 62 AND 69 AND 75) | 197 |
| 77 | PsycINFO | 76 [DT 2000-2020] [Peer reviewed] [Languages English] | 110 |
| 78 | EMBASE | (suicid*).ti,ab | 95198 |
| 79 | EMBASE | (44 OR 78) | 209671 |
| 80 | EMBASE | (33 AND 37 AND 51 AND 79) | 227 |
| 81 | EMBASE | 80 [DT 2000-2020] [Priority journals] [Publication types Article OR Journal OR Review] [English language] | 33 |
| 82 | CINAHL | (prison* OR inmate* OR offender* OR convict*).ti,ab | 17946 |
| 83 | CINAHL | PRISONERS/ | 9566 |
| 84 | CINAHL | exp "PUBLIC OFFENDERS"/ | 11830 |
| 85 | CINAHL | (82 OR 83 OR 84) | 28525 |
| 86 | CINAHL | (staff OR officer* OR governor* OR nurse* OR physician* OR employee* OR doctor*).ti,ab | 652013 |
| 87 | CINAHL | exp "CORRECTIONAL HEALTH SERVICES"/ | 1878 |
| 88 | CINAHL | exp "CORRECTIONAL FACILITIES PERSONNEL"/ | 446 |
| 89 | CINAHL | exp "HEALTH PERSONNEL"/ | 586687 |
| 90 | CINAHL | (86 OR 87 OR 88 OR 89) | 1039283 |
| 91 | CINAHL | (self AND (wound* OR mutilat* OR harm* OR injury OR injuries)).ti,ab | 20427 |
| 92 | CINAHL | exp "INJURIES, SELF-INFLICTED"/ | 2812 |
| 93 | CINAHL | "RISK FOR SELF-MUTILATION (NANDA)"/ OR exp "RISK FOR VIOLENCE, SELF-DIRECTED OR DIRECTED AT OTHERS (NANDA)"/ | 13 |
| 94 | CINAHL | "SELF MUTILATION RISK (SABA CCC)"/ OR "SUICIDE RISK (SABA CCC)"/ | 3 |
| 95 | CINAHL | (suicid*).ti,ab | 33625 |
| 96 | CINAHL | SUICIDE/ | 19326 |
| 97 | CINAHL | (auto mutilation OR automutilation).ti,ab | 24 |
| 98 | CINAHL | (DSH OR deliberate self harm*).ti,ab | 825 |
| 99 | CINAHL | (cutting).ti,ab | 8175 |
| 100 | CINAHL | (91 OR 92 OR 93 OR 94 OR 95 OR 96 OR 97 OR 98 OR 99) | 64555 |
| 101 | CINAHL | (attitude* OR perception* OR experience* OR opinion* OR understand* OR perspective* OR view* OR feel*).ti,ab | 1015701 |
| 102 | CINAHL | exp "ATTITUDE OF HEALTH PERSONNEL"/ OR ATTITUDE/ | 120378 |
| 103 | CINAHL | "EMPLOYEE ATTITUDES"/ | 4821 |
| 104 | CINAHL | (COMPREHENSION OR comprehend).ti,ab | 11271 |
| 105 | CINAHL | exp EMOTIONS/ | 140927 |
| 106 | CINAHL | exp "BEHAVIOR AND BEHAVIOR MECHANISMS"/ | 1803377 |
| 107 | CINAHL | (101 OR 102 OR 103 OR 104 OR 105 OR 106) | 2319380 |
| 108 | CINAHL | (85 AND 90 AND 100 AND 107) | 169 |
| 109 | CINAHL | 108 [DT 2000-2020] [Peer reviewed] [Languages eng] | 148 |
| 110 | Medline | 29 [DT 2000-2020] [Languages English] | 100 |
| 111 | EMBASE | 80 [DT 2000-2020] [English language] | 172 |
| 112 | PsycINFO | 76 [DT 2000-2020] | 162 |
| 113 | CINAHL | 108 [DT 2000-2020] [Languages eng] | 156 |
| 114 | PsycINFO | 76 [DT 2000-2020] [Languages English] | 153 |
| 115 | Medline | 29 [DT 2000-2020] | 113 |
| 116 | EMBASE | 80 [DT 2000-2020] | 187 |
| 117 | CINAHL | 108 [DT 2000-2020] | 160 |
| 118 | PsycINFO | 76 [DT 2000-2020] | 162 |
| 119 | Medline | (8 OR 9 OR 10 OR 11 OR 12 OR 13 OR 15) | 130909 |
| 120 | Medline | (7 AND 24 AND 28 AND 119) | 140 |
| 121 | Medline | 120 [DT 2000-2020] | 112 |
| 122 | EMBASE | (38 OR 39 OR 40 OR 41 OR 42) | 135827 |
| 123 | EMBASE | (78 OR 122) | 170341 |
| 124 | EMBASE | (33 AND 37 AND 51 AND 123) | 225 |
| 125 | EMBASE | 124 [DT 2000-2020] | 185 |
| 126 | PsycINFO | (63 OR 64 OR 65 OR 66 OR 67) | 86467 |
| 127 | PsycINFO | (57 AND 62 AND 75 AND 126) | 195 |
| 128 | PsycINFO | 127 [DT 2000-2020] | 160 |
| 129 | CINAHL | (92 OR 93 OR 94 OR 95 OR 96 OR 97 OR 98) | 40846 |
| 130 | CINAHL | (85 AND 90 AND 107 AND 129) | 122 |
| 131 | CINAHL | 130 [DT 2000-2020] | 115 |

**Appendix 2 – Grey literature search strategy**

**Google scholar**

Search terms: Self-harm AND prison AND staff AND (attitudes OR perceptions OR experiences)

Search strategy: The first 100 results of this search engine were reviewed and screened by title, abstract and full-text.

**Google**

Search terms: Self-harm AND prison AND staff AND (attitudes OR perceptions OR experiences)

Search strategy: The first 100 results of this search engine were reviewed and screened by title, abstract and full-text.

**Open Grey**

Search terms: Self-harm AND prison

Search strategy: All outputs (n=10) were reviewed and screened by title, abstract and full-text

**Websites**

Websites searched: Ministry of Justice, The Howard League, Independent Advisory Panel on Deaths in Custody, Prison Reform Trust

Search strategy: All websites were hand-searched to identify any relevant links or articles, which were then screened by title, abstract and full-text

**Appendix 3: Record of studies excluded from the systematic review**

| **Authors (Year)** | **Title** | **Reason for exclusion** |
| --- | --- | --- |
| Auzolt & Abdellaoui  (2013) | Perceptions of a peer suicide prevention program by inmates and professionals working in prisons | Lack of focus on self-harm |
| Barker, Kolves & De Leo  (2014) | Management of suicidal and self-harming behaviors in prisons: systematic literature review of evidence-based activities | Study type (systematic review) |
| Bennett & Dyson  (2014) | Deliberate self-harm among adults in prisons | Study type (systematic review) |
| Borrill & Taylor  (2009) | Suicides by foreign national prisoners in England and Wales in 2007: mental health and cultural issues | Lack of relevance – does not include the attitudes, experiences or perceptions of prison staff |
| Bowers, Carr-Walker, Paton, Nijman, Callaghan, Allen, & Alexander (2006) | Changes in attitudes to personality disorder on a DSPD unit | Lack of relevance – focussed on personality disorder rather than self-harm specifically. |
| Camilleri & McArthur  (2008) | Suicidal behaviour in prisons: Learning from Australian and international experiences. | Lack of relevance – does not include the attitudes, experiences or perceptions of prison staff towards self-harm |
| Carr-Walker, Bowers, Callaghan, Nijman, & Paton (2004) | Attitudes towards personality disorders: Comparison between prison officers and psychiatric nurses | Lack of focus on self-harm |
| Crighton & Towl | Forensic psychology (book) | Study type |
| Danks & Bradley  (2018) | Negotiating barriers: prisoner and staff perspectives on mental wellbeing in the open prisons setting | Lack of focus on self-harm |
| Dear (2008) | Ten years of research into self-harm in the Western Australian prison system: Where to next? | Lack of relevance to staff attitudes, perceptions and experiences |
| Dickinson, Wright & Harrison  (2009) | The attitudes of nursing staff in secure environments to young people who self-harm | Study location and sample – takes place in secure environments (not prisons) and focuses on self-harm performed by young people rather than adults |
| Eccleston & Sorbello  (2002) | The RUSH program – Real understanding of Self-help: A suicide and self-harm prevention initiative within a prison setting | Lack of relevance – focuses on a specific self-harm prevention initiative, with a lack of focus on the attitudes, experiences, or perceptions of prison staff towards self-harm |
| Elger, Handtke & Wangmo  (2015) | Paternalistic breaches of confidentiality in prison: mental health professionals’ attitudes and justifications | Lack of relevance – focuses on breaches of confidentiality in prison including in relation to suicidality, but not on the attitudes, perceptions, or experiences of prison staff towards self-harm |
| Fillmore & Dell  (2003) | Violence and self-harm among women in conflict with the law | Study population – includes staff working in community settings (outside of prisons) |
| Fillmore, Dell & Elizabeth Fry Society of Manitoba  (2000) | Prairie women, violence, and self-harm | Study population – includes staff working in community settings (outside of prisons) |
| Foster, Bell & Jayasinghe  (2013) | Care control and collaborative working in a prison hospital | Lack of focus on self-harm |
| Griffiths & Bailey  (2015) | Learning from peer support schemes – can prison listeners support offenders who self-injure in custody? | Lack of relevance - focuses on staff perceptions of peer support for self-harm rather than self-harm itself |
| Hall & Gabor  (2004) | Peer suicide prevention in a prison | Lack of relevance – focuses on attitudes towards the Listeners scheme rather than attitudes towards self-harm itself |
| Hayes, Shaw, Lever-Green, Parker & Gask  (2008) | Improvements to suicide prevention training for prison staff in England and Wales | Lack of relevance – focussed on evaluation of a specific training package for suicide prevention rather than the attitudes, perceptions and experiences of prison staff relating to self-harm |
| Jewkes (2007) | Handbook on Prisons | No original research reported |
| Lewis, Lewis & Garby  (2013) | Surviving the trenches: The personal impact of the job on probation officers. | Study population – probation officers who may or may not have worked in prisons |
| MacDonald  (2013) | Women prisoners, mental health, violence and abuse | Lack of focus on self-harm |
| Maltman & Hamilton  (2011) | Preliminary evaluation of personality disorder awareness workshops for prison staff | Lack of focus on self-harm |
| Marzano & Alder  (2007) | Supporting staff working with prisoners who self-harm: A survey of support services for staff dealing with self-harm in prisons in England and Wales | Lack of relevance – focused on reported provision of staff support services rather than attitudes, perceptions and experiences of self-harm |
| Musselwhite, Freshwater, Jack, & Maclean (2004) | Mental health awareness for prison staff | Lack of relevance to staff attitudes, perceptions, and experiences |
| Nicolas et al (2012) | Banged up bandaged up a qualitative study of non-suicidal self-injury amongst young women in prison | Study setting – conducted in young offender institutions |
| Powell, Harris, Condon & Kemple  (2010) | Nursing care of prisoners: staff views and experiences | Lack of focus on self-harm |
| Perry et al  (2019) | Implementation of a problem-solving training initiative to reduce self-harm in prisons: a qualitative perspective of prison staff, field researchers and prisoners at risk of self-harm. | Lack of relevance - focuses on attitudes towards a specific self-harm training initiative rather than self-harm itself |
| Piccoli, Pizzighello & Tolio  (2015) | Level of burnout and aptitude toward suicide in penitentiary health care staff | No full text available (published as an e-poster) |
| Pope  (2018) | Self-harm by adult men in prison: A rapid evidence assessment (REA) | Study type – this is a rapid evidence assessment and does not report original research |
| Riding, Swann & Swann  (2005) | The handbook of forensic learning disabilities | Study setting – forensic hospitals and not prisons |
| Sheeran  (2012) | Suicide prevention training in a county jail | Lack of relevance – focuses on staff attitudes towards suicide prevention training rather than self-harm or suicide itself |
| Slade & Forrester (2015) | Shifting the paradigm of prison suicide prevention through enhanced multi-agency integration and cultural change | Lack of relevance - lack of focus on the attitudes, experiences, or perceptions of prison staff towards self-harm |
| Snow & McHugh (2002) | The aftermath of a death in prison custody | Lack of original research |
| Snoyman, Aicken, Ware & Spilsbury  (2013) | Staff use of mandatory notification as a means of reducing suicide and self-harm in NSW correctional centres | Lack of focus on self-harm |
| Towl & Forbes (2002) | Working with suicidal prisoners | No original research reported |
| Woodfield, Boduszek & Willmott (2019) | Introduction and psychometric validation of the prison personnel trauma measure (PPTM) | Lack of specific focus on self-harm |

**Appendix 4: Quality assessment ratings of individual studies**

| **Authors (Year)** | **Title** | **Tool Used** | **Rating** |
| --- | --- | --- | --- |
| Bantjes, Swartz & Niewoudt (2017) | Human rights and mental health in post-apartheid South Africa: lessons from health care professionals working with suicidal inmates in the prison system | CASP | Poor |
| Callahan (2004) | Correctional officer attitudes toward inmates with mental disorders | NIH | Poor |
| Cassidy & Bruce  (2019) | Dealing with death in custody: psychosocial consequences for correctional staff | NIH | Moderate |
| Cresswell, Karimova & Ward  (2018) | Women, self-harm, and the moral code of the prison | MMAT | Poor |
| DeHart, Smith & Kaminski (2009) | Institutional Responses to Self-Injurious Behavior Among Inmates | MMAT | Moderate |
| Garbutt & Casey (2015) | Attitudes towards prisoners who self harm scale: A psychometric evaluation | NIH | Moderate |
| Howard League for Penal Reform  (2017) | Preventing prisoner suicide: staff perspectives | CASP | Poor |
| Ireland & Quinn (2007) | Officer Attitudes Towards Adult Male Prisoners Who Self-Harm: Development of an Attitudinal Measure and Investigation of Sex Differences | NIH | Moderate |
| Kenning et al (2010) | Prison staff and women prisoner’s views on self-harm; their implications for service delivery and development: A qualitative study | CASP | Good |
| Ludlow et al  (2015) | Self-inflicted deaths in NOMS’ custody in 18-24-year olds: Staff experience, knowledge and views | CASP | Good |
| Liebling et al  (2005) | An evaluation of the safer locals programme: Final report | MMAT | Good |
| Marzano  (2007) | Self-harm in a men’s prison: staff’s and prisoner’s perspectives | CASP | Good |
| Marzano, Alder & Ciclitira (2013) | Responding to repetitive, non-suicidal self-harm in an English male prison: Staff experiences, reactions, and concerns | CASP | Moderate |
| Moore, Andargachew & Taylor (2010) | Working with women prisoners who seriously harm themselves: Ratings of staff expressed emotion (EE) | CASP | Moderate |
| Pannell, Howells & Day (2003) | Prison Officer’s Beliefs Regarding Self-Harm in Prisoners: An Empirical Investigation | NIH | Moderate |
| Ramluggun (2013) | A critical exploration of the management of self-harm in a male custodial setting: qualitative findings of a comparative analysis of prison staff views on self-harm | CASP | Moderate |
| Rivlin (2010) | Suicide and self-injurious behaviours at HMP Grendon | CASP | Poor |
| Rivlin (2007) | Self-harm and suicide at Grendon Therapeutic Community Prison | CASP | Poor |
| Short et al (2009) | Custody vs care: attitudes of prison staff to self-harm in women prisoners—a qualitative study | CASP | Good |
| Slade & Lopresti  (2013) | Promoting resilience in prison staff | NIH | Poor |
| Smith et al (2019) | Working with prisoners who self‐harm: A qualitative study on stress, denial of weakness, and encouraging resilience in a sample of correctional staff | CASP | Good |
| Smith & Kaminski | Self-injurious behaviors in state prisons | NIH | Poor |
| Sousa, Gonçalves, Cruz & Rodrigues (2019) | Prison officers' attitudes towards self-harm in prisoners | NIH | Poor |
| Sweeney, Clarbour & Oliver (2018) | Prison officers’ experiences of working with adult male offenders who engage in suicide-related behaviour | CASP | Moderate |
| Tait (2011) | A typology of prison officer approaches to care | CASP | Moderate |
| Walker et al (2017) | Coping with the job’: prison staff responding to self-harm in three English female prisons: a qualitative study | CASP | Moderate |
| Walker et al (2017) | Supporting imprisoned women who self-harm: exploring prison staff strategies | CASP | Moderate |
| Ward (2014) | Women’s imprisonment, self-harm and emancipatory research: Developing a framework for transformative research in a women’s prison | MMAT | Good |
| Ward & Bailey (2011) | At arms length: the development of a self-injury training package for prison staff through service user involvement | MMAT | Moderate |
| Ward & Bailey (2013) | A participatory action research methodology in the management of self-harm in prison | MMAT | Moderate |
| Wood-Schultz (2012) | Variability in personal characteristics among professional staff providing suicide prevention responses in correctional settings | NIH | Poor |
| Wright, Borrill, Teers & Cassidy (2006) | The mental health consequences of dealing with self-inflicted death in custody | NIH | Moderate |
